# Usefulness of open mixed nut challenges to exclude tree nut allergy in children

**DOI:** 10.1186/s13601-015-0062-y

**Published:** 2015-05-16

**Authors:** Francine C. Van Erp, André C. Knulst, Irene L. Kok, Maartje F. van Velzen, Cornelis K. van der Ent, Yolanda Meijer

**Affiliations:** Department of Paediatric Pulmonology and Allergology, Wilhelmina Children’s Hospital, University Medical Centre Utrecht, 3508 AB, P O Box 85090, Utrecht, The Netherlands; Department of Dietetics, Internal Medicine and Dermatology, University Medical Centre Utrecht, Utrecht, The Netherlands; Department of (Paediatric) Dermatology and Immunlogy, University Medical Centre Utrecht, Utrecht, The Netherlands

**Keywords:** Tree nuts, Nut allergy, Diagnostics, Food challenge

## Abstract

**Background:**

To minimize the risk of accidental reactions, atopic children with multiple sensitizations to tree nuts are advised to avoid all nuts. Multiple food challenges would be needed to confirm the clinical relevance, but are too burdensome to be practical.

The usefulness of open mixed nut challenges in terms of safety, reactions during challenge, tolerance of the challenge material, effect on the elimination diet and satisfaction of the parents was evaluated.

**Findings:**

Open mixed nut challenges were performed in 19 children with a previous negative hazelnut challenge and long term elimination diet for tree nuts. Challenges were negative in 13 (68 %) children, in four (21 %) children (non-severe) allergic symptoms were observed. The challenges were well accepted, safe and efficient. We were able to avoid multiple nut challenges in 15 (79 %) children.

**Conclusions:**

Open mixed nut challenge can efficiently exclude multiple tree nut allergies in children with a lifelong nut free diet and low suspicion of clinical allergy.

## Findings

Suspected food allergy based on sensitisation without known ingestion often results in elimination diets and subsequent social isolation, fear of anaphylaxis and a decreased quality of life [1]. In atopic children tree nut elimination diets are often advised due to multiple tree nut sensitisations together with severe eczema or other (severe) food allergies [2, 3]. In those children introduction at home is often not possible due to the possible risk of allergic reactions and fear of the parents. The Double Blind Placebo Controlled Food Challenge (DBPCFC) is the gold standard to diagnose food allergy [4]. In children presenting with a (life) long elimination diet for tree nuts, multiple challenges would be necessary to rule out the presence of relevant tree nut allergies. This is however difficult in practice as DBPCFCs are time consuming, expensive and burdensome procedures. Open mixed nut challenges have previously been shown to efficiently discriminate between multiple tree nut allergies and a single nut allergy [5]. In this study we describe our experience with open mixed nut challenges as a diagnostic tool to exclude multiple nut allergies in children with long term elimination diets for tree nuts.

We performed a retrospective case note review of food challenges performed in our tertiary food allergy clinic from 2012-2014 and selected children who underwent a mixed nut challenge. Data were obtained as part of regular clinical care, collected from electronic patient records by their responsible clinician and used in strictly anonymous form, according to the code of conduct for medical research approved by the hospital’s Medical Ethical Committee.

Nineteen children with a previous negative hazelnut challenge and a lifelong preventive elimination diet for multiple tree nuts underwent a mixed nut challenge as part of clinical care. Children without a history of tree nut related symptoms and with low levels of sIgE to all tree nuts were challenged with six nuts (almond, walnut, cashew, pistachio, pecan, Brazil nut) (protocol A), Children with suspected cashew/pistachio allergy (previous symptoms or a specific IgE to cashew or pistachio of > 10 kU/L) were challenged with four nuts (almond, walnut, pecan, Brazil nut) (protocol B). In short, 5 g of each tree nut was blended and mixed with apple sauce. Increasing amounts of the mixture were given in an open challenge with 21 grams of whole tree nuts as the last step in the protocol (Table 1). Challenges were discontinued and considered positive in case of objective symptoms or if suggestive subjective symptoms occurred at 3 subsequent doses or a subjective symptom lasted for more than 45 minutes. In case of a negative challenge parents were advised to introduce all tested tree nuts subsequently into the diet of their child. After positive or inconclusive challenges an expert team of allergists decided whether (guided) reintroduction of tree nuts was possible or future multiple single nut challenges were indicated at the patient’s request. One month after challenge parents were contacted by phone to evaluate the reintroduction of tree nuts, dietary restrictions and satisfaction about the challenge procedures.

**Table 1:** Challenge protocol of mixed nut challenges

			Mixture A^a^	Mixture B^b^
Portion	Time (min)	Mixture dose (g)	Tree nuts dose (g)	Tree nuts dose (g)
1	0	1	0,2	0,1
2	15	4	0,7	0,5
3	30	11	2,2	1,5
4	60	33	6,7	4,4
5	90	100	20,1	13,3
6	150	Open challenge with whole nuts (2 almonds, 2 half walnuts,2 half pecans, 1 Brazil nut, 2 cashew nuts*, 2 pistachios*)	ca. 21	ca. 17
		Total	51	37

* Only in mixture A

^a^Mixture A: 5 g of each nut (almond, walnut, cashew, pistachio, pecan, Brazil nut) and 120 g apple sauce.

^b^Mixture B: 5 g of each nut (almond, walnut, pecan, Brazil nut) and 130 g apple sauce.

Measurement of specific IgE (sIgE) was performed in all children within 1 year prior to the food challenge, using Immuno CAP-technique (Phadia, Uppsala, Sweden), IgE levels of ≥ 0.35 kU/L were considered positive. The presence of asthma, atopic dermatitis, allergic rhinitis and other food allergies was determined in out-patient clinic consultations before food challenge. Reactions during the challenge, tolerance of the nut mix challenge material, and satisfaction of the parents were evaluated with descriptive statistics.

Children who underwent the mixed nut challenge had a mean age of 10.1 (range: 5.4-17.1) years. Elimination diets for tree nuts were based on (multiple) sensitizations together with known other food allergy in 14 (74 %) or severe eczema in 3 (16 %) children. Two other children (12 %) eliminated tree nuts based on a history of immediate symptoms after ingestion of cashew or pistachio. Challenges were performed with mixture B in four children. Individual characteristics and sensitization patterns of all children are shown in Table 2.

**Table 2:** Children who underwent a mixed nut challenge

Case	Age (yrs)	Reason for long elimination diet	Mix	Max dose (g)*	Symptoms	Outcome	Conclusion & advice	Pre diet	Post diet	sIgE Grass	sIgE Birch	sIgE Haz	sIgE Alm	sIgE Cas	sIgE Pis	sIgE Bra	sIgE Pec	sIgE Wal
1	6,5	Multiple sensitization and peanut allergy	A	6,7	Aversion with OAS, abdominal pain, vomiting	+/−	Tree nut allergy, multiple challenges	No nuts	No nuts	0,5	1,5	13,1	1,1	3,0	1,2	0,4	0,6	0,4
2	6,6	Multiple sensitization and reaction to 1 nut (Cas S2)	B	13,3	Aversion	+/−	Suspected cas/pis allergy introduction of alm, bra, pec, wal	Alm	Alm, bra, pec, wal^1^	0,2	0,0	0,9	0,0	1,1	0,2	0,0	0,0	0,0
3	15,2	Multiple sensitization and eczema	A	20,1	Rhino-conjunctivitis	+	Suspected tree nut allergy, introduction of alm, bra, pec	No nuts	Alm, bra, pec^1^	10,4	76,0	21,8	0,3	1,2	2,1	0,1	0,3	7,1
4	9,7	Multiple sensitization and peanut allergy	A	6,7	Rash, urticaria, conjunctivitis, sensation of throat tightness, abdominal pain	+	Tree nut allergy, multiple challenges	No nuts	Alm, wal^2^	19,6	37,0	15,5	2,7	7,9	7,8	1,5	0,3	1,2
5	9,4	Multiple sensitization and other food allergy	A	2,2	OAS, rhino-conjunctivitis, vomiting	+	Tree nut allergy, multiple challenges	No nuts	No nuts	50,0	101,0	74,0	0,4	2,7	4,1	0,2	0,3	0,3
6	5,4	Multiple sensitization and other food allergy	A	6,7	Rash, rhino-conjunctivitis, angioedema, vomiting	+	Tree nut allergy, multiple challenges	Alm	Alm	1,7	5,0	2,3	0,2	2,3	3,3	0,0	0,5	0,6
7	8	Multiple sensitization and other food allergy	A			-	Introduction all nuts	Alm	None	37,0	73,0	32,8	0,5	0,0	0,0	0,1	0,1	0,0
8	11,4	Multiple sensitization and reaction to 1 nut (Pis S5)	B			-	Introduction tested nuts	Ntn	No nuts^3^	0,0	0,0	2,0	0,6	12,0	23,7	0,4	0,7	2,9
9	6,3	Multiple sensitization and other food allergy	B			-	Introduction tested nuts	Ntn	No nuts^4^	14,1	101,0	19,7	0,3	7,3	10,8	0,0	0,8	2,8
10	15,4	Multiple sensitization and peanut allergy	A			-	Introduction all nuts	Ntn	No nuts^5^	101,0	43,0	8,5	2,4	0,4	0,6	0,1	0,9	1,9
11	10	Multiple sensitization and peanut allergy	A			-	Introduction all nuts	No nuts	None	7,9	2,3	2,3	2,5	1,1	1,6	1,0	1,8	2,1
12	9	Multiple sensitization and peanut allergy	A			-	Introduction all nuts	No nuts	None	56,0	101,0	87,0	7,0	1,2	1,0	0,9	0,7	0,3
13	9,8	Multiple sensitization and eczema	A			-	Introduction all nuts	No nuts	None	11,3	1,4	0,5	0,3	0,3	0,6	0,2	0,2	0,2
14	9,7	Multiple sensitization and peanut allergy	A			-	Introduction all nuts	No nuts	None	101,0	101,0	101,0	7,4	1,6	2,5	1,2	2,9	2,6
15	8	Multiple sensitization and eczema	A			-	Introduction all nuts	No nuts	No nuts^6^	19,0	17,8	8,6	0,3	0,8	1,0	0,4	0,0	0,1
16	17,1	Multiple sensitization and peanut allergy	A			-	Introduction all nuts	Ntn	None	16,0	36,0	27,9	2,3	1,8	1,2	1,1	0,2	0,4
17	14,1	Multiple sensitization and other food allergy	A			-	Introduction all nuts	No nuts	None	4,4	13,2	7,2	0,3	0,1	0,2	0,1	0,2	0,3
18	10	Multiple sensitization and peanut allergy	A			-	Introduction all nuts	Ntn	None	17,3	28,3	18.90	1,4	0,6	0,8	0,4	0,1	0,1
19	12,5	Multiple sensitization and peanut allergy	B			-	Introduction tested nuts	No nuts	No nuts^4^	30,0	101,0	3.50	1,0	17,4	20,7	0,5	1,2	2,1

Alm, almond; Bra, Brazil nut; Cas, cashew nut; Haz, hazelnut; Ntn, no traces of nuts; Pec, pecan; Pis, pistachio; Wal, walnut

* Maximum dose in total grams mixed tree nuts

+, positive; −, negative; +/−, inconclusive

^1^Introduction started based on negative sIgE results

^2^Introduction started by parents

^3^Introduction not started due to comorbidities of the child

^4^Introduction successful but preventive nut free diet due to possible cashew or pistachio allergy

^5^Oral allergy symptoms after whole nuts

^6^Introduction not started, parents are used to diet due to other food allergies

In 13 children (68 %) the mixed nut challenge was considered negative. Allergic symptoms were observed in 4 (21 %) children, all with Sampson grade 3 reactions [6]. No correlation between levels of sIgE and outcome of the mixed nut challenge could be found. In one positive tested child (case 3), IgE negative nuts were introduced successfully. In one other child (case 4) parents successfully introduced almond and walnut based on their own decision. In 2 (11 %) young children the test was inconclusive due to aversion. In one of those children (case 2) tree nuts with negative sIgE results were successfully introduced at home without additional challenges. Overall we were able to avoid multiple challenges in 15 (79 %) children and successfully could expand the diet in 14 (74 %) children (Table 2). All parents were satisfied about the challenge material, challenge protocol and outcome of the challenge.

We successfully performed mixed nut challenges in children with previous negative hazelnut challenge and low suspicion of (multiple) tree nut allergy. Hazelnut allergy was ruled out first because sIgE to hazelnut was relatively high (mean 24.5 kU/L). Moreover, hazelnut is one of the most frequently consumed tree nuts in Europe and the most prevalent cause of tree nut allergy in the Netherlands [7]. No clear cut-off point of sIgE to tree nuts was used to select children for mixed nut challenge as the results of the few previous published studies on this topic differ widely between patient populations and tree nuts [8, 9]. However, children were never challenged when any sIgE level to tree nuts was above 10 kU/L. The lack of correlation between sIgE values and mixed nut challenge outcome in our study demonstrates that the usefulness of multiple allergen testing in children with lifelong elimination diets is questionable. In children without multiple tree nut sensitization and without previous symptoms after ingestion, introduction at home is justified [4]. Nevertheless, in case of extreme anxiety in children and/or parents, mixed nut challenges were useful and successfully performed for this reason in three children (cases 7,13 and 17). Children with a clear history of tree nut related symptoms were excluded or not tested for cashew/pistachio as they were expected to develop symptoms during the mixed nut challenge and therefore were unlikely to benefit from the mixed nut challenge in terms of accurate diagnosis and opportunities to expand the diet. In those children single nut challenges with nuts relevant to the child’s diet were performed at parent’s request. When symptoms occurred during challenge the true diagnostic value of the mixed nut challenge remained questionable as the culprit nut was unknown. However, as proposed by Ball et al. previously, it is debatable whether single nut challenges will change the outcome of advice in children with (multiple) tree nut allergy as products are often not labelled with individual nuts [5]. Moreover even after negative mixed nut challenge other reasons as other food allergies or habituation to the diet (patient 15) can prevent patients from introducing tree nuts. Nonetheless, our data show that most parents (74 %) do manage to introduce (single) nuts in the diet even when aversion and or allergic symptoms are present during mixed nut challenge.

When interpreting the results of this study some limitations have to be discussed. We performed mixed nut challenges in a small and selected patient population to exclude multiple tree nut allergies and diminish unnecessary dietary restrictions in children with long term elimination diets. Further research is necessary to determine whether mixed nut challenges are feasible in different populations. We did not have information on skin prick tests or sensitization to tree nut components. In the future, it might be possible to exclude tree nut allergy at least for some tree nuts based on component resolved diagnostics [10]. However, some parents will not introduce tree nuts at home because of fear for an allergic reaction, in those cases mixed nut challenge are still useful despite its limited diagnostic value. Previously published data provide evidence for a high degree of cross reactivity between walnut and pecan and cashew and pistachio [11, 12]. As a consequence one could argue that mixed nut challenges can be simplified to contain four nuts only (cashew or pistachio, walnut or pecan, almond and Brazil nut). The challenge material of our mixed nut challenges is simple and can be easily implemented in daily practice and adapted to the individual patient. It is unclear whether the material in its present form is suitable for children below the age of six. As an alternative, a biscuit with mixed nuts was used successfully in a previous study [5]. This however requires a specialized kitchen and involves heating of the allergens which can reduce their allergenicity [13]. All parents were satisfied with the challenge, however in future research it would be important to compare our results with parents of children who underwent multiple single challenges.

In conclusion, our data show that open mixed nut challenges are useful to exclude multiple tree nut allergies in children in with a lifelong nut free diet and low suspicion of clinical allergy. In those children mixed nut challenges can prevent multiple single nut challenges and help to facilitate introduction of tree nuts at home, even when symptoms during challenge occur.

## Abbreviations

DBPCFC: Double blind placebo controlled food challenge
SIgE: Specific IgE
